# Prostate and urinary microbiomes in prostate cancer development: focus on *Cutibacterium acnes*

**DOI:** 10.3389/fcimb.2025.1562729

**Published:** 2025-05-21

**Authors:** Fangzhi Fu, Yunfeng Yu, Biao Wang, Xiang Zhao, Neng Wang, Jubo Yin, Kai Wu, Qing Zhou

**Affiliations:** Department of Andrology, The First Affiliated Hospital of Hunan University of Chinese Medicine, Changsha, China

**Keywords:** prostate cancer, microbiome, bacteria, *Cutibacterium acnes*, urine

## Abstract

Prostate cancer (PCa) is one of the most prevalent malignancies among men, with its incidence steadily increasing worldwide. Recent advances in microbiome research have opened new avenues for understanding and treating PCa; however, studies focusing specifically on the prostate tissue microbiome remain limited. Evidence suggests that the microbial communities within PCa tissues exhibit significant diversity and regional variability, with certain bacteria potentially contributing to PCa initiation and progression through chronic inflammation. The prostate microbiome comprises not only bacteria but also viruses, fungi, and parasites, and its diversity is influenced by a complex interplay of genetic, environmental, and lifestyle factors. Methodological limitations and sample contamination further complicate the interpretation of microbiome data. The urinary microbiome is similarly diverse and shaped by multiple overlapping influences. Although urine, prostatic fluid, and prostate tissue are anatomically and functionally connected, whether urine and prostatic fluid can accurately reflect the prostate tissue microbiome remains to be conclusively determined. Among the microorganisms detected, *Cutibacterium acnes* is frequently identified in prostate tissue, urine, and prostatic fluid from PCa patients. This bacterium is known to elicit inflammatory responses through various pathways, potentially impacting tumorigenesis and cancer progression. Nevertheless, findings across studies remain inconsistent. Further research is necessary to elucidate the underlying mechanisms by which the microbiome influences PCa. Such efforts may offer novel insights and strategies for the diagnosis, treatment, and prevention of this disease.

## 1 Introduction

### 1.1 Contemporary clinical settings and treatment approaches

Prostate cancer (PCa) is one of the most prevalent malignancies in men, with a steadily increasing global incidence. According to recent statistics, PCa currently ranks as the most commonly diagnosed malignancy among men in the United States (Miller et al., 2022). Mutations in the androgen receptor (AR) gene are a hallmark of disease progression, with nearly all patients eventually developing castration-resistant prostate cancer (CRPC). At advanced stages, bone metastases frequently occur, significantly impairing patients’ quality of life (Coleman et al., 2020; Tilki et al., 2024). The underlying mechanisms of PCa bone metastasis remain incompletely understood. Tumor-derived factors such as extracellular vesicles, inflammatory chemokines, and interactions between cancer cells and the extracellular matrix (ECM) are believed to contribute to metastatic progression. Pain and skeletal-related events, particularly fractures, are major determinants of decreased quality of life in affected patients (Kang et al., 2022). Current European guidelines recommend a personalized, risk-adapted approach to PCa screening and treatment. Risk stratification plays a central role in determining appropriate therapeutic strategies, which may include active surveillance, radical prostatectomy, androgen deprivation therapy, radiotherapy, and immunotherapy (Adamo et al., 2024; Cornford et al., 2024; Kumar Am et al., 2024). Multidisciplinary collaboration is emphasized to enhance treatment efficacy and improve patient prognosis. Despite these advancements, significant challenges remain. High recurrence rates and treatment resistance continue to limit long-term outcomes (Almeeri et al., 2024). These challenges underscore the complexity of PCa and highlight the need for more comprehensive and patient-centered multimodal treatment strategies aimed at improving both survival and quality of life (Ahmadzadehfar et al., 2024). In recent years, the role of the human microbiome in cancer biology has received growing attention. Preliminary evidence suggests that the microbiome may influence cancer initiation, progression, and metastasis (Fares et al., 2020; Herrera-Quintana et al., 2024). A deeper understanding of the interactions between the microbiome and PCa may provide novel insights and therapeutic targets, potentially leading to more effective treatment paradigms and better clinical outcomes.

### 1.2 The microbiome as a new frontier in precision cancer therapy

The human body hosts a diverse and dynamic array of microorganisms, including bacteria, viruses, fungi, and archaea, collectively referred to as the microbiome (Zuber et al., 2023). This complex ecosystem plays a crucial role in maintaining physiological homeostasis, and its composition is highly individualized and shaped by genetic, environmental, and lifestyle factors over time. In recent years, the microbiome has emerged as a promising area of research in the field of precision oncology (El Tekle and Garrett, 2023). In the context of PCa, microbiome research primarily focuses on two domains: the microbiomes of prostate tissue and urine, and those of the oral cavity and gastrointestinal tract (Pernigoni et al., 2023; Shyanti et al., 2024). While studies on the oral and gut microbiomes are relatively abundant, investigations into the microbial communities in prostate tissue and urine remain limited. In particular, evidence concerning the prostate tissue microbiome is still insufficient and warrants further exploration (Munteanu et al., 2023). The oral and gut microbiomes represent highly complex microbial environments that influence host immune responses, metabolic regulation, and systemic inflammation (Helmink et al., 2019). Strong evidence linking gut microbiota dysbiosis to cancer development is primarily concentrated in colorectal cancer (Tilg et al., 2018). Specific bacterial species have been identified as contributors to colorectal tumorigenesis, with microbial imbalance disrupting the equilibrium between pro-tumorigenic pathogens and anti-tumor commensals (Wong and Yu, 2023). Emerging research suggests that alterations in the gut microbiome may also play a role in the pathogenesis and therapeutic responsiveness of PCa. These effects are thought to be mediated through mechanisms involving modulation of androgen metabolism, immune regulation, and intestinal barrier integrity (Romano et al., 2024; Zha et al., 2023). Therefore, targeting the gut microbiome and its metabolites represents a novel and potentially transformative strategy for the diagnosis and treatment of prostate-related diseases.

**Figure 1:**
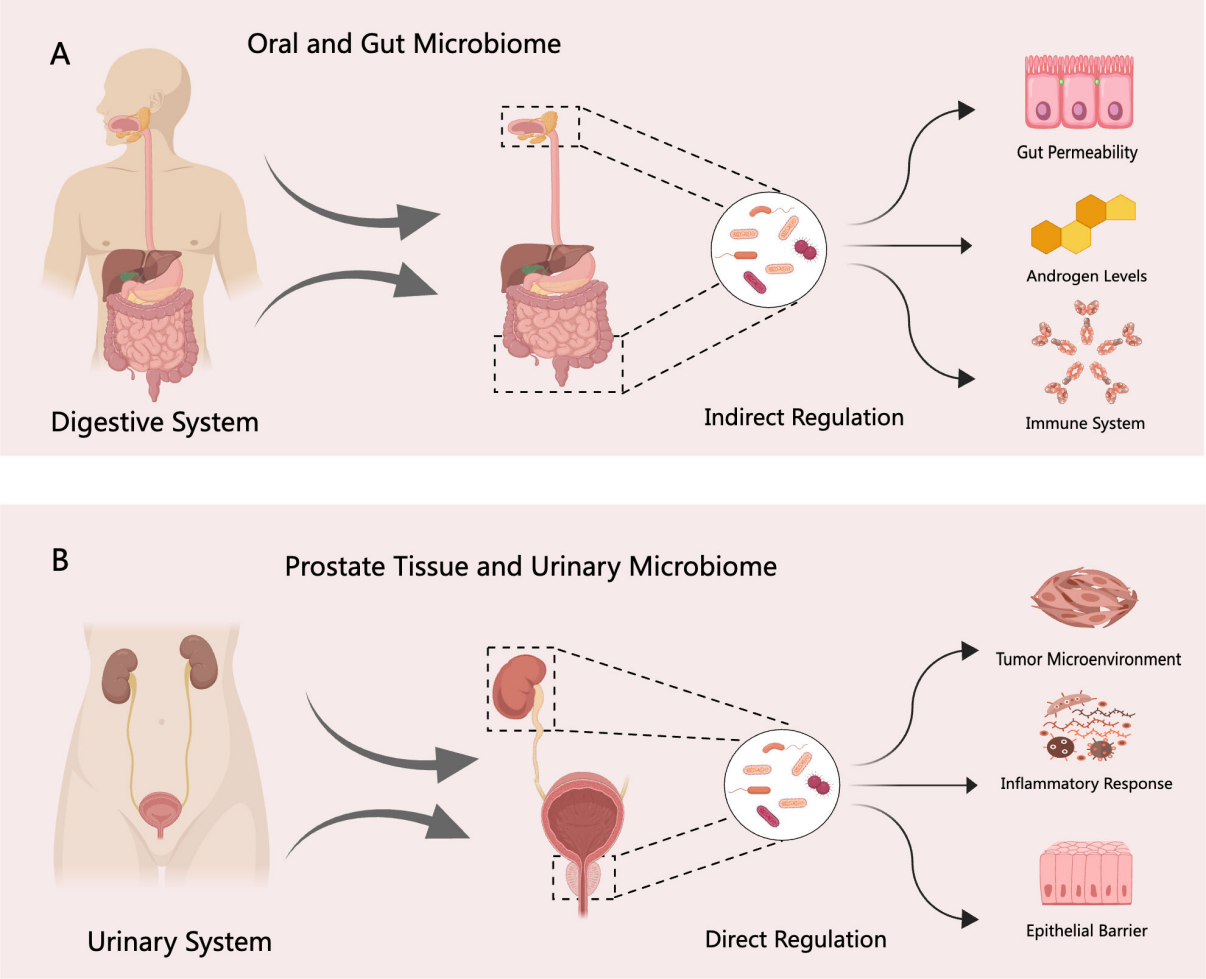
Regulatory mechanisms of the digestive and urinary system microbiomes in PCa. **(A)** The digestive system microbiome—including oral and gut microbial communities—may indirectly influence the initiation and progression of PCa through several pathways, such as disruption of the intestinal barrier, modulation of androgen levels, and regulation of host immune responses. **(B)** In contrast, the urinary system microbiome—comprising prostate tissue and urinary microbial communities—may directly contribute to PCa pathogenesis by altering the tumor microenvironment, inducing inflammatory responses, and impairing epithelial barrier integrity.

Unlike the gut microbiome, the urinary tract microbiome primarily comprises microbial communities in the prostate tissue and urine (Tsai et al., 2022). Although direct evidence linking these microbiomes to clinical outcomes in PCa is currently limited compared to the extensive research on the gut microbiome (Mjaess et al., 2023), emerging studies suggest their potential roles in modulating cancer treatment resistance and immune responses within the tumor microenvironment. For example, tumor-resident *Proteobacteria* have been shown to alter the metabolism of chemotherapeutic agents such as gemcitabine, thereby contributing to drug resistance in pancreatic cancer (Geller et al., 2017). Moreover, intratumoral microbiota can both enhance and suppress host immune responses, ultimately influencing the efficacy of immunotherapies (Pushalkar et al., 2018; Zheng et al., 2017). Given these findings, in-depth investigation into the prostate tissue and urine microbiomes is essential to unravel the complex interactions between microbial populations and PCa pathophysiology. A conceptual framework of these interactions and potential regulatory mechanisms is illustrated in **Figure 1**. Such research may pave the way for novel therapeutic strategies. Promising avenues include antibiotic regimens, fecal microbiota transplantation, immunotherapy, and molecular targeted therapies (Dicks and Vermeulen, 2022; Rizzo et al., 2022).

## 2 Diversity and regional differences of the PCa microbiome

### 2.1 The relationship between bacteria and inflammation in PCa

Growing evidence suggests that the prostate microbiota plays a potentially significant role in the initiation, progression, and prognosis of PCa within the tumor microenvironment (Ohadian Moghadam and Momeni, 2021). While the use of bacteria from the tumor microenvironment for targeted therapy holds promise, comprehensive experimental validation is still lacking (Che et al., 2021). Historically, the prostate was believed to be a sterile organ; however, subsequent studies have challenged this notion (Krieger et al., 2000; Leskinen et al., 2003). Although bacteria are typically absent in prostate tissues from healthy individuals, both bacterial presence and inflammation have been observed in radical prostatectomy specimens. These findings suggest that microbial colonization may contribute to localized inflammatory microenvironments, potentially playing a role in prostate pathology (Hochreiter et al., 2000).

Prostate inflammation can arise from various sources, including microbial infections (e.g., sexually transmitted pathogens), cellular injury (e.g., urine reflux, prostatic calculi), hormonal fluctuations, and environmental exposures (De Marzo et al., 2007). These stimuli may trigger local tissue damage and immune activation, resulting in a breakdown of immune tolerance to self-antigens within the prostate. Chronic inflammation, in turn, may foster a microenvironment conducive to carcinogenesis. Early investigations using 16S rRNA gene sequencing revealed that bacterial species detected in localized PCa were not specific to chronic prostatitis (Keay et al., 1999). Subsequently, Banarjee et al. employed microarray-based metagenomic analysis and found no statistically significant differences in microbial composition between PCa and benign prostatic hyperplasia (BPH) patients (Banerjee et al., 2019). Similarly, Feng et al., using an integrated approach combining metagenomics and metatranscriptomics, reported no clear association between the prostate microbiome and metastatic progression in PCa (Feng et al., 2019b).

However, recent studies have presented alternative perspectives regarding the role of the prostate microbiome in PCa. Ma et al. were the first to utilize microbial data from TCGA to comprehensively investigate the potential pro-tumorigenic and anti-tumorigenic roles of the prostate microbiota in PCa (Ma et al., 2020). Their analysis revealed a correlation between thermophilic bacteria and *Streptococcus pneumoniae* with PSA levels, suggesting a potential involvement in the inflammatory microenvironment of PCa. Additionally, several bacterial taxa were negatively associated with Gleason scores, TNM staging, and AR expression. These findings were further supported by the work of Guner et al., who demonstrated that chronic inflammation of the prostate, associated with microbial presence, was linked to a higher risk of Gleason score upgrading and increased tumor aggressiveness (Guner et al., 2021).

**Figure 2:**
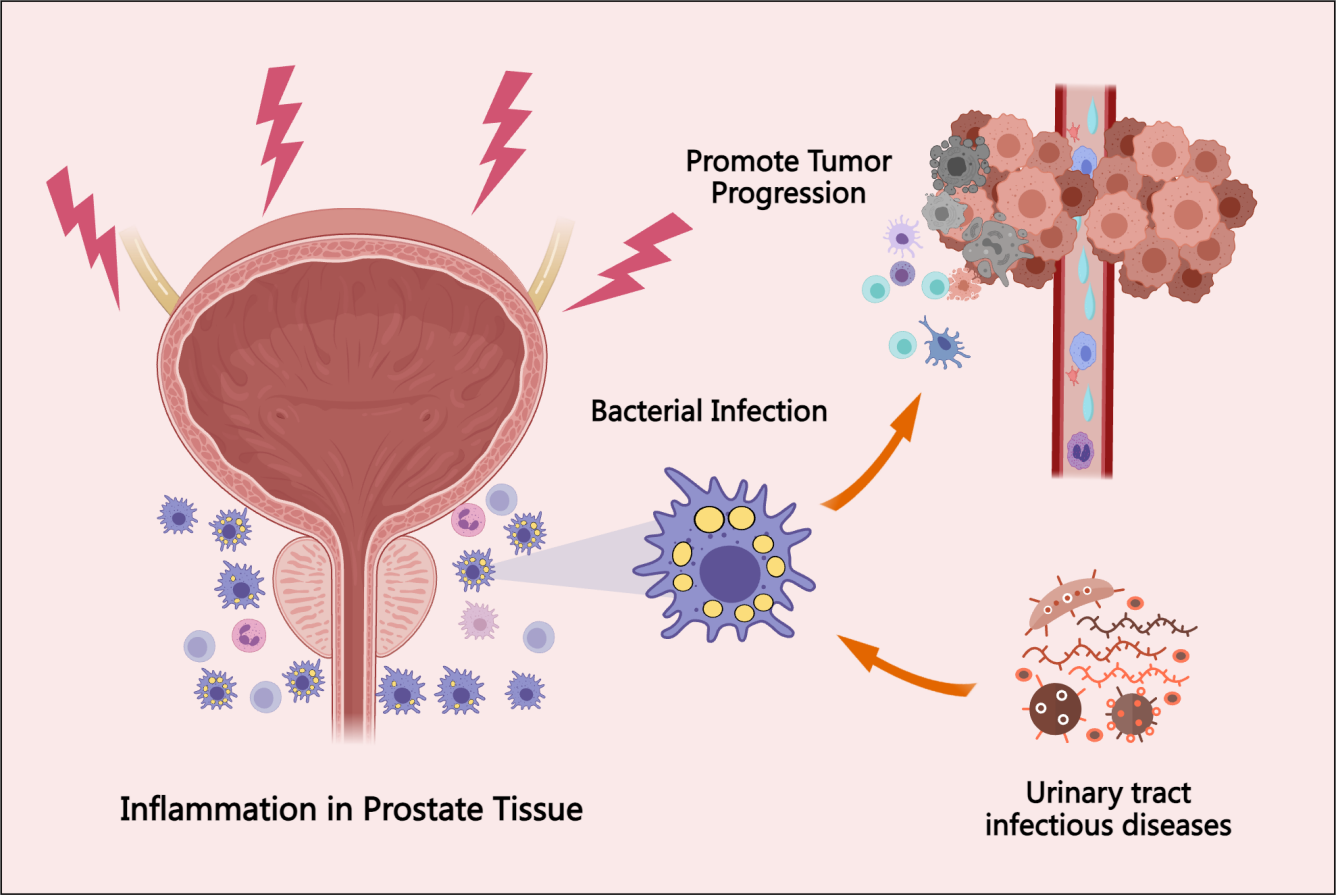
The association between prostate tissue bacterial infection, inflammatory responses, and PCa progression. Bacterial infections in the prostate may originate from urinary tract infections, local tissue injury, hormonal fluctuations, and environmental exposures. These infections elicit localized inflammatory responses, which can promote tumor initiation and progression. This figure highlights the involvement of the prostate microbiome in the underlying mechanisms and pathophysiological processes of PCa.

While microbial infections may indeed contribute to inflammation within the prostate microenvironment, it is important to recognize that prostate inflammation is a multifactorial process. The direct relationship between the prostate microbiome and inflammation remains insufficiently characterized (Sfanos et al., 2018). Moreover, certain bacterial sequences may exist within prostate tissues that are not detectable through conventional culture methods or standard sequencing technologies, making accurate identification challenging (Riley et al., 1998). Recent evidence has also highlighted associations between pathogenic microbiota—particularly those involved in urinary tract infections (UTIs) and vesicoureteral reflux—and PCa development (Prakash et al., 2024). The prostate microbiome, which originates from factors such as urinary tract infections and local trauma, may contribute to the progression of prostate cancer (PCa) through the induction of localized inflammatory responses (Picardo et al., 2019). The interaction between microbiome-induced inflammation and the pathogenesis of PCa is illustrated in **Figure 2**.

### 2.2 Bacterial community composition and differential analysis in PCa tissues

As sequencing technologies have advanced, whole-genome and metagenomic sequencing based on next-generation sequencing (NGS) platforms are increasingly employed to investigate the composition and variations of prostate tissue microbiota between PCa patients and healthy controls (Lloréns-Rico et al., 2022). Although these techniques are now widely utilized in human microbiome studies, they present distinct limitations and technical challenges. For example, while 16S rRNA gene sequencing is cost-effective and widely accessible, it often fails to detect low-abundance microbial taxa, especially those that are difficult to culture under standard laboratory conditions. Additionally, many of these low-abundance species are underrepresented in metagenomic reference databases (Almeida et al., 2021). In contrast, metagenomics allows for comprehensive, unbiased sequencing of all genetic material within a sample, offering higher taxonomic resolution down to the species and even strain level. However, metagenomic analysis is more expensive, computationally demanding, and particularly susceptible to host DNA contamination—an issue that is especially pronounced in low-biomass environments such as the prostate and urinary tract (Wensel et al., 2022). Early studies using 16S rRNA sequencing technology provided initial insights into the prostate microbiome. Cohen et al. reported bacterial culture positivity in 56% of PCa tissue samples, identifying organisms such as *C. acnes* and coagulase-negative staphylococci (Cohen et al., 2005). Similarly, Sfanos et al. analyzed core prostate tissue samples from 30 cancer patients and found *Escherichia* spp., *Acinetobacter* spp., and *Pseudomonas* spp. to be the most frequently detected bacterial taxa (Sfanos et al., 2008). Notably, these bacteria were present in 95% of analyzed samples, but considerable regional heterogeneity in bacterial composition was observed among different cores from the same individual—potentially reflecting localized variations in the tissue microenvironment. Building on these findings, Yow et al. applied both 16S rRNA sequencing and total RNA sequencing to prostate tissue and identified *Escherichia* spp. and *Cutibacterium acnes* as predominant species, primarily from the *Enterobacteriaceae* family (Yow et al., 2017). Cavarretta et al. performed a comparative analysis of tumor, tumor-adjacent, and non-tumor prostate tissues, revealing a microbiome dominated by *Actinobacteria*, followed by *Firmicutes* and *Proteobacteria* (Cavarretta et al., 2017). At the genus level, *Staphylococcus* spp. were more abundant in tumor and adjacent tissues, whereas *Streptococcus* spp. were more prevalent in non-tumorous regions, suggesting intra-organ microbial heterogeneity that may be associated with PCa pathogenesis. Further supporting these observations, Banerjee et al. used microarray-based metagenomic analysis and found high relative abundances of *Proteobacteria*, *Firmicutes*, *Actinobacteria*, and *Bacteroidetes* in PCa tissues (Banerjee et al., 2019). These results were consistent with earlier findings by Sfanos and Yow. Feng et al., using an integrative approach combining metagenomics and metatranscriptomics, reported significant enrichment of *Escherichia* spp., *Acinetobacter* spp., *Pseudomonas* spp., and *C. acnes* in PCa tissues (Feng et al., 2019b). In a separate metatranscriptomic study, Salachan et al. confirmed the high relative abundance of *Acinetobacter*, *Enterobacter*, *Streptococcus*, and *Escherichia* in prostate tissue samples from PCa patients (Salachan et al., 2022).

Although these studies have shed light on the characteristics of the prostate microbiome in PCa patients, notable inconsistencies remain across the findings. For example, Salachan et al. reported a marked decrease in *Bacteroides fragilis*, *Staphylococcus saprophyticus*, and *Vibrio parahaemolyticus*, alongside an abnormal increase in *Sphingomonas*, further enriching the pool of potential microbial candidates associated with PCa progression (Salachan et al., 2022). In another study, Sarkar et al. utilized 16S rRNA sequencing to compare microbiome differences between patients with BPH and PCa (Sarkar et al., 2022). They found that *Prevotella*, *Cupriavidus*, and *C. acnes* were the most abundant bacterial genera in prostate lesions. LEfSe analysis identified *Cupriavidus* and *Methylobacterium* as significantly enriched in PCa samples, whereas *Corynebacterium* and *Fibrobacter* were more abundant in BPH samples. Gonçalves et al. also applied 16S rRNA sequencing and observed significant compositional differences between PCa and normal prostate samples, with lower microbial species richness detected in PCa tissues (Gonçalves et al., 2023). In PCa samples, *Alistipes*, *Sutterella*, *Klebsiella*, and *Rothia* were relatively enriched, while *Actinomyces*, *Bacteroides*, *Prevotella*, and *Muribaculum* predominated in normal tissues. However, contrasting results were reported by Kim et al., who found no significant differences in microbiome composition or diversity between PCa patients with biochemical recurrence and those without recurrence (Kim et al., 2023). Nevertheless, LEfSe analysis suggested a potential link between biochemical recurrence and the presence of *Lactobacillus*, which may represent a prognostic marker for PCa recurrence. In a follow-up study, Kim et al. expanded their analysis using 16S rRNA sequencing and reported that *Proteobacteria*, *Bacteroidetes*, and *Firmicutes* were the predominant phyla in PCa samples. At lower taxonomic levels, *Bacteroidales*, *Clostridiales*, *Burkholderiales*, and *Rhizobiales* were the most common bacterial orders, while *Ruminococcaceae*, *Comamonadaceae*, *Rhizobiaceae*, and *Enterococcaceae* were the most represented families (Kim et al., 2024). Other studies have yielded similar disparities. For example, Lee et al. found no significant differences in microbial diversity or composition between PCa patients and healthy controls using 16S rRNA sequencing (Lee et al., 2024). However, their analysis indicated that *Sutterella*, *Escherichia*, *Delftia*, and *Gordonia* were more abundant in normal prostate tissues, whereas *Sphingomonas*, *Peptostreptococcus*, *Sphingobacterium*, and *Enterobacter* were more prevalent in PCa tissues. Although various studies have identified associations between specific bacterial genera and PCa, consistent diagnostic or prognostic biomarkers have yet to be established (Crocetto et al., 2020). The observed heterogeneity in microbial profiles and their correlations with PCa across different cohorts underscores the need for further in-depth and standardized investigations. Elucidating the complex and dynamic relationship between the prostatic microbiome and PCa remains a critical step toward understanding its pathophysiological relevance and potential clinical utility.

### 2.3 The impact of race and environmental factors on the bacterial characteristics of PCa

Ethnic and environmental factors appear to play a significant role in shaping the prostate microbiome and may influence PCa progression. Feng et al. investigated the effects of ethnicity (African vs. European ancestry) and geographic environment (African vs. Australian cohorts) on the prostate microbiome. Their analysis revealed that African samples exhibited significantly greater microbial diversity, aligning with previous findings from China. These samples were predominantly composed of *C. acnes*, *Escherichia coli*, and *Pseudomonas* spp., with *Proteobacteria* identified as the dominant phylum in PCa microbiomes. Compared to European samples, African specimens showed increased species richness, potentially correlating with a higher tumor mutation burden (Feng et al., 2019a). Additionally, genera such as *Bacteroides*, *Firmicutes*, *Prevotella*, and *Fusobacterium* were detected exclusively in African samples. While such differences may reflect true biological variation, the potential for sample contamination must be carefully considered. Notably, nearly half of the core human gut bacterial taxa were absent in these datasets. As reported by Feng, Salachan, and others, no consistent correlation has been observed between the prostate microbiome and local PCa progression. This may be attributable to the low microbial biomass typically present in prostate tissues, as well as the risk of contamination during sampling and sequencing procedures. Chen et al. further highlighted these issues by demonstrating that bacterial DNA yields from biopsy-derived prostate tissues were markedly lower than those obtained from urine samples or skin swabs (Chen et al., 2024). Their findings also showed that *Lactobacillus* and *Staphylococcus* were highly abundant in perineal regions, reinforcing the need to rigorously exclude potential skin and environmental contaminants when analyzing prostate tissue microbiomes.

Contaminant bacteria introduced during sampling or laboratory procedures may be mistakenly identified as constituents of the prostate microbiome, a well-recognized limitation in current research. In Salachan et al.’s sequencing study, seven of the ten most abundant microorganisms were classified as likely laboratory contaminants. Only *Salmonella enterica*, *Campylobacter jejuni*, and *Clostridium difficile* were considered potential true microbiota, although these species are typically associated with the gastrointestinal tract (Salachan et al., 2022). Similarly, previous studies have identified genera such as *Acinetobacter*, *Enterobacter*, *Streptococcus*, *Escherichia*, *Bacillus*, *Mycobacterium*, *Pseudomonas*, and *Staphylococcus* as common sources of contamination (Eisenhofer et al., 2019). These microorganisms may originate from external sources, adjacent tissue cross-contamination, or the laboratory environment, potentially confounding microbiome profiling and interpretation. Therefore, strict contamination control protocols are essential for obtaining accurate and reliable results. While some of these microbes may indeed be involved in PCa pathogenesis, their specific roles remain unclear and require further investigation.

To address concerns regarding contamination, Chen et al. proposed an innovative approach utilizing transperineal biopsy for prostate microbiome analysis. This technique circumvents the potential introduction of gut microbiota that often accompanies traditional transrectal biopsy methods (Chen et al., 2024). Beyond procedural improvements, researchers have also emphasized optimizing experimental design to reduce the influence of confounding factors on microbiome profiling. For instance, Lee et al. employed 64 negative control samples to rigorously eliminate potential contaminant sequences, thereby minimizing bias in sequencing-based analyses. Their findings indicated that the microbiome composition in PCa patients was affected by comorbid diabetes, suggesting that metabolic conditions may modulate the prostate tissue microenvironment via microbiome alterations (Lee et al., 2024). These efforts reflect a growing awareness among researchers regarding the impact of contamination and the importance of continuous methodological refinement. Moreover, pharmacological interventions may also influence PCa risk and progression by modulating microbial composition. Certain medications can suppress pro-inflammatory bacteria or promote the enrichment of beneficial microbes (Barone et al., 2023; Crocetto et al., 2023). For instance, long-term antibiotic use has been linked to persistent dysbiosis in both gut and urogenital microbiota. These disruptions may indirectly affect the immune status and microecological balance of the prostate, potentially contributing to tumorigenesis and influencing the characteristics of the tumor microenvironment in PCa.

### 2.4 Viral, fungal, and parasitic characteristics in the PCa microbiota

The prostate is a biologically complex and dynamic organ that can harbor mixed microbial infections, including not only bacteria but also viruses, fungi, and parasites. Several viruses with established or suspected oncogenic potential have been detected in prostate tissue, such as BK virus (BKV), JC virus (JCV), Simian Virus 40 (SV40), Xenotropic Murine Leukemia Virus-related virus (XMRV), Human Papillomavirus (HPV), and Human Cytomegalovirus (HCMV) (Samanta et al., 2003; Zambrano et al., 2002). In a study by Martinez-Fierro et al., HPV, XMRV, and HCMV were detected in PCa tissues. Notably, a positive association was observed between HPV infection and PCa, while BKV, JCV, and SV40 were not detected (Martinez-Fierro et al., 2010). In addition to bacterial analysis, Banerjee et al. performed a comprehensive pan-pathogen microarray metagenomic study to investigate viral, fungal, and parasitic diversity in PCa tissues. Their results indicated that herpesviruses and papillomaviruses—specifically HCMV, HPV16, and HPV18—were more prevalent in cancer cohorts. Fungal species from the phylum *Ascomycota*, such as dermatophytes and *Candida* spp., were commonly identified. Among parasitic taxa, nematodes such as *Hookworm* and *Ascaris* were the most frequently detected (Banerjee et al., 2019). Importantly, HPV16 and HPV18—both high-risk oncogenic HPV genotypes—are considered potential contributors to PCa pathogenesis. Miyake et al. also detected HPV16, HPV18, and *Mycoplasma genitalium* in PCa cohorts (Miyake et al., 2019). Similarly, Sarkar et al. confirmed a significant association between HPV16, HPV18, and PCa, while also implicating Epstein-Barr virus (EBV) and Hepatitis B virus (HBV) as potential microbial signatures relevant to PCa (Sarkar et al., 2022).

In contrast, Feng et al. did not detect viruses, fungi, or archaea in their metagenomic analysis, instead identifying only bacterial taxa (Feng et al., 2019a). Ala-Almohadesin et al. employed quantitative TaqMan real-time PCR to detect sexually transmitted infection (STI) pathogens in prostate tissues from patients with BPH and PCa. Their results indicated that only *Gardnerella vaginalis* (GV) and Herpes Simplex Virus 2 (HSV-2) were significantly associated with PCa, while no significant differences were observed for HPV, HCMV, or other herpesviruses (Ala-Almohadesin et al., 2019). Similarly, Gonçalves et al. did not detect typical STI-associated bacteria in their PCa cohort (Gonçalves et al., 2023). These findings suggest that not all oncogenic pathogens are directly involved in PCa, or that their effects may be mediated indirectly through chronic inflammation. In other studies, Salachan et al. reported that the abundance of *Haemophilus* β-herpesvirus was significantly lower in malignant tissues compared to benign counterparts (Salachan et al., 2022). Ethnic and geographic variations have also been observed in viral profiles. For example, Chen et al. found SV40, HCMV, and EBV in Chinese patient samples, whereas these viruses were absent in samples from Western patients (Chen and Wei, 2015). These disparities suggest that the diversity and distribution of PCa-associated microbiota may be influenced by ethnicity, geography, and individual host factors. Further research is essential to elucidate the roles of these non-bacterial microbes in prostate carcinogenesis and their potential as diagnostic or therapeutic targets. A summary of these findings is presented in **Table 1**.

**Table 1:** Characteristics of the prostate tissue microbiome.

Researcher	Year	Technique	Subjects	Key Findings
Cohen et al.	2005	16S rRNA sequencing technology	34 PCa patient prostate tissue samples	19 samples were positive for bacterial culture, 12 detected C. acnes, with others including coagulase-negative staphylococci; specific C. acnes subtypes may contribute to prostate inflammation.
Sfanos et al.	2008	16S rRNA sequencing technology	170 prostate tissue samples and 200 patient DNA samples from 30 PCa patients	Escherichia, Acinetobacter, and Pseudomonas were the most common bacterial taxa in the prostate microbiome; bacterial distribution showed regional heterogeneity in different tissue core samples, with no significant association between bacteria and inflammation.
Martinez-Fierro et al.	2010	TaqMan genotyping technology	Prostate tissue samples from 55 PCa patients and 75 healthy men	HPV was detected in 11 PCa samples and 4 normal samples, with a positive correlation between PCa and HPV infection; some samples had multiple HPV subtypes.
Yow et al.	2017	16S rRNA sequencing and total RNA sequencing technology	20 prostate tissue samples from 10 PCa patients	All samples contained members of the Enterobacteriaceae family, with Escherichia and C. acnes being the most abundant; C. acnes was detected in 95% of the samples.
Cavarretta et al.	2017	High-throughput pyrosequencing technology	Prostate tissue samples from 16 PCa patients (tumor, peritumoral, and non-tumor)	The prostate microbiome was dominated by Actinobacteria, followed by Firmicutes and Proteobacteria; at the genus level, C. acnes was the most abundant; Staphylococcus was common in tumor and peritumoral tissues, while Streptococcus had higher abundance in non-tumor tissues.
Banarjee et al.	2019	Microarray metagenomics analysis technology	Prostate tissue samples from 50 PCa patients and 15 BPH patients	Microbial profiles differed between the two groups; Proteobacteria, Firmicutes, Ascomycota, and Nematoda were more abundant in PCa patients; Herpesviridae and Papillomaviridae were more prevalent in PCa samples; 85% of PCa samples tested positive for C. acnes, Chlamydia trachomatis, Mycoplasma, HCMV, and HPV1.
Feng et al.	2019	Metagenomics and metatranscriptomics technology	Tumor and adjacent benign prostate tissue samples from 65 PCa patients	C. acnes, Escherichia, Acinetobacter, and Pseudomonas were significantly enriched in PCa prostate tissues; Pseudomonas infection may hinder PCa metastasis; no association was found between the microbiome and local progression of PCa.
Feng et al.	2019	Metagenomics technology	Prostate tissue samples from 6 African PCa patients and 16 European PCa patients	All samples were enriched with Proteobacteria, with the most abundant genera being Escherichia, C. acnes, and Pseudomonas; African samples showed higher species richness compared to European samples, with enrichment in Streptococcus, Sphingobium, Acidovorax, and Escherichia; Bacteroides, Propionibacterium, Parabacteroides, and Odoribacter were present only in African samples.
Miyake et al.	2019	PCR Detection technology	Prostate tissue samples from 45 PCa patients and 33 BPH patients	Compared to BPH samples, PCa samples had higher infection rates of Ureaplasma urealyticum, HPV16, and HPV18; no significant correlation was found between Ureaplasma urealyticum infection status and prostate inflammation grade; Ureaplasma urealyticum infection was associated with younger age.
Ala-Almohadesin et al.	2019	TaqMan Real-Time PCR technology	Prostate tissue samples from 180 PCa patients and 63 BPH patients	The highest detection rate of Atopobium vaginae was found in both groups, followed by Ureaplasma urealyticum; compared to BPH samples, the infection rates of GV and HSV-2 were lower in PCa samples.
Salachan et al.	2022	Meta-transcriptomics analysis technology	Discovery cohort: 106 prostate tissue samples from 94 PCa patients; Validation cohort: 24 prostate tissue total RNA sequencing results from a public dataset (8 benign and 16 malignant)	Higher relative abundance of genera such as Acinetobacter, Enterobacter, Streptococcus, and Escherichia in prostate tissue; a significant decrease of Bacillus fragilis, Staphylococcus saprophyticus, and Vibrio parahaemolyticus in PCa samples, with an abnormal increase of Shewanella; compared to T2 tumors, the species of Mycobacterium increased in pathological T3 tumors.
Sarkar et al.	2022	16S rRNA sequencing technology	Discovery cohort: 33 PCa and 13 BPH prostate tissue samples; Validation cohort: 16 PCa and 15 BPH prostate tissue samples	A significant decrease in species richness in PCa samples, with no significant difference in bacterial composition between BPH and PCa samples; at the phylum level, Proteobacteria was most abundant in PCa, while Actinobacteria significantly decreased; at the genus level, Prevotella, Cupriavidus, C. acnes, Acinetobacter, and Bacillus were the five most abundant genera in PCa; a significant association between HPV-16 and HPV-18 and PCa; Taiwan Cupriavidus and methylotrophic Methylobacterium increased in PCa, while Wetland Kurthia and mixed Bacillus megaterium were enriched in BPH samples.
Gonçalves et al.	2023	16S rRNA sequencing technology	15 PCa patients and 15 normal males’ prostate tissue samples	Significant differences in microbiota composition between PCa and normal samples, with lower species richness in PCa samples; in PCa samples, Alishewa, Veillonella, Klebsiella, and Rothia were more abundant; in normal samples, Actinobacteria, Bifidobacterium, Prevotella, and Muribaculum were more abundant.
J. H. Kim et al.	2023	16S rRNA sequencing technology	13 biochemical recurrence and 13 non-recurrence PCa patients’ prostate tissue samples	No significant differences in microbiota composition and richness between the two groups; biochemical recurrence samples had a higher total bacterial count, while non-recurrence samples had more Lactobacillus.
J. H. Kim et al.	2024	16S rRNA sequencing technology	11 low-grade and 15 high-grade PCa patients’ prostate tissue samples	No significant differences in microbiota composition and richness between the two groups; Firmicutes was the most abundant phylum. Compared to low-grade samples, high-grade samples had a higher abundance of Actinobacteria at the phylum level, higher abundance of Actinobacteria at the class level, higher abundance of Propionibacteriales at the order level, higher abundance of Propionibacteriaceae and Bacillaceae at the family level; and higher abundance of Bacillus at the genus level.
Chen et al.	2024	16S rRNA sequencing technology	32 PCa patients and 10 normal male prostate tissue, urine, and perineal samples	Significant differences in microbiota composition between perineal, urine, and prostate tissue samples; no significant differences in microbiota composition and richness between PCa and normal male samples; Pseudomonas was relatively more abundant in PCa samples; Bacillus and Staphylococcus were more abundant in perineal samples.
J.-J. Lee et al.	2024	16S rRNA sequencing technology	59 PCa patients and 59 normal male prostate tissue samples	No significant differences in microbiota composition and richness between the two groups; Sphingomonas, Peptoniphilus, Shewanella, and Enterobacter were more abundant in PCa samples; Pseudomonas, Escherichia, Delftia, and Gordonia were more abundant in normal samples; The relative abundance of C. acnes was lower in the high pathology group compared to the intermediate pathology group.

## 3 The potential value of urine and prostate fluid microbiota in PCa prediction

Recent research supports the utility of urine as a non-invasive source for identifying biomarkers predictive of PCa (Nardelli et al., 2024; Zemskova et al., 2020). Tsai et al., using 16S rRNA sequencing, demonstrated distinct differences in the urinary microbiota compositions of patients with BPH and PCa (Tsai et al., 2022). Specifically, *Lactobacillus* and *Staphylococcus* exhibited statistically significant variation between these patient groups and healthy controls. In a large-scale cohort study, Pan et al. reported a significant association between PCa and UTIs, including prostatitis, cystitis, and pyelonephritis. This association implies increased susceptibility of PCa patients to alterations in urinary microbiota, particularly those linked to infection and inflammation (Pan et al., 2023). Supporting this, Prakash et al. proposed that pathogen-associated microbiota involved in UTIs may contribute to PCa development. For instance, bladder-ureteral reflux could facilitate pathogen migration and induce prostatic inflammation, underscoring the need for early diagnosis and close microbial monitoring of recurrent UTIs (Prakash et al., 2024). The urogenital microbiome is relatively well characterized and lends itself to repeated sampling over time. Studies have shown that urinary microbiota predominantly belong to five major phyla: *Firmicutes*, *Bacteroidetes*, *Actinobacteria*, *Clostridia*, and *Proteobacteria* (Siddiqui et al., 2011). Shrestha et al. found that the urine microbiota in men with both positive and negative PCa biopsies was typically dominated by a single bacterial genus, most commonly *Bacillus*, *Staphylococcus*, or *Streptococcus* (Shrestha et al., 2018). Other commonly detected genera include *Lactobacillus* and *Prevotella* (Kustrimovic et al., 2023). However, notable differences in bacterial abundance and composition have been observed between voided urine and prostate tissue. Since urine samples also contain microbiota from the bladder and urethra, even midstream urine collection may not accurately reflect the true microbial landscape of the prostate. This raises important concerns regarding the reliability of urine as a surrogate for studying the prostate microbiome (Kim et al., 2022).

Due to variations in sample selection, anatomical source, and procedural techniques, discrepancies persist among studies investigating the urinary and prostate fluid microbiota in PCa. For example, Hurst et al. analyzed urine and prostate tissue samples collected after digital rectal examination and identified four novel bacterial species commonly present in patient urine—*Porphyromonas*, *Bacteroides*, *Vagococcus*, and *Fusobacterium*. Furthermore, specific anaerobic bacterial communities detected in urine were associated with higher PCa risk, as classified by the D’Amico criteria, suggesting potential prognostic relevance (Hurst et al., 2022). Alanee et al. performed high-throughput 16S rRNA gene sequencing on urine samples from 30 patients with suspected PCa and found that microbial alterations were primarily driven by changes in bacterial abundance rather than diversity. Notably, *Lactobacillus* abundance decreased, while *Streptococcus* abundance increased in PCa patients (Alanee et al., 2019). In a follow-up study, Lee et al. reported the presence of bacterial toxin genes—including colicin, cytotoxic necrotizing factor, and cytolytic pore-forming toxins—in the urine of high-grade PCa patients. However, these toxins were not considered diagnostically significant and were instead hypothesized to be associated with prior infections or environmental exposures (Lee et al., 2023). In African cohorts, Akinpelu et al. observed higher pathogen isolation rates in PCa samples compared to BPH, with *Escherichia coli* identified as the most prevalent pathogen, followed by *Pseudomonas aeruginosa* (Akinpelu et al., 2024). Microbiota identified in urinary microbiome studies may originate from various anatomical sites, including the prostate, bladder, and glans penis. Yu et al. conducted 16S rRNA sequencing on urine, expressed prostatic secretions (EPS), and semen from PCa and BPH patients. In PCa patients, EPS samples showed an increased abundance of *Bacteroidetes*, *Proteobacteria*, and *Firmicutes*, along with significant decreases in anaerobic genera such as *Selenomonas* and *Deinococcus*. Quantitative real-time PCR revealed decreased counts of *E. coli* and *Enterococcus* in urine, but increased abundance in prostatic fluid and semen (Yu et al., 2015). In another study, Ma et al. analyzed EPS microbiota using 16S rRNA sequencing and reported reduced microbial diversity in PCa patients. *Streptococcus*, *Bacillus*, *Enterobacteriaceae*, and *Lactococcus* were more abundant in the EPS of PCa patients, whereas *Marinobacter* and *Thermobacterium* were more prevalent in non-PCa controls (Ma et al., 2019). Gonçalves et al. compared microbiota from urine and the glans penis and found their compositions to be highly similar. In the urine of PCa patients, elevated levels of *Streptococcus*, *Prevotella*, and *Peptostreptococcus* were observed, while *Methylobacterium*, *Bacteroides*, and *Clostridium* were more abundant in controls (Gonçalves et al., 2023). Collectively, these findings demonstrate significant variability in microbial profiles across different urogenital sampling sites in PCa, as detailed in **Table 2**. Further research is needed to determine whether the observed microbial differences in urine and prostate fluid represent true biological signatures and whether they correlate with clinical outcomes.

**Table 2:** Characteristics of the Urine and Prostate Fluid Microbiome.

Researcher	Year	Technique	Subjects	Key Findings
Yu et al.	2015	16S rDNA sequencing, quantitative real-time PCR analysis technology	Urine, prostatic fluid, and semen samples from 13 PCa patients and 20 BPH patients	Significant differences in the microbial composition between the urine samples of PCa and BPH; compared to BPH, Bacteroidetes, Proteobacteria, and Firmicutes were increased in the PCa urine samples, while Anaerococcus, Flavonifractor, Escherichia coli, and Enterococcus were significantly reduced; in PCa semen and prostatic fluid samples, Escherichia coli and Enterococcus were significantly increased.
Shrestha et al.	2018	16S rRNA sequencing technology	Urine samples from 70 PCa patients and 63 healthy male controls	No significant differences in microbial composition or richness between the two groups; the urine microbiome in both groups was predominantly dominated by a single genus, such as Bacillus, Staphylococcus, and Streptococcus; PCa urine samples contained several pro-inflammatory bacteria, including Actinomyces and Ureaplasma.
Alanee et al.	2019	16S rRNA sequencing technology	Urine samples from 14 PCa patients and 16 healthy male controls	The differences in the urine microbiome between the two groups were mainly due to changes in microbial richness rather than composition; in PCa urine samples, the abundance of bacteria such as Veillonella, Streptococcus, and Bacteroides increased, while the abundance of species like Bifidobacterium, Lactobacillus, and Actinobacteria decreased.
Ma et al.	2019	16S rRNA sequencing technology	Prostatic fluid samples from 32 PCa patients and 27 healthy male controls	The microbiome richness in PCa prostatic fluid samples was lower, with no significant differences in the microbiome composition between the two groups; in PCa prostatic fluid samples, the relative abundance of Streptococcus, Alkalibacterium, Enterobacter, and Lactococcus was higher; in normal prostatic fluid samples, the relative abundance of Oceanobacillus and Thermoactinomyces was higher.
Hurst et al.	2022	16S rRNA, RNA-seq, whole genome DNA sequencing, and anaerobic culturing techniques	Urine samples from 318 PCa patients	The presence of bacteria in urine sediments was significantly associated with higher D’Amico risk groups for PCa; four new bacterial species were identified in the urine, originating from the Firmicutes, Actinobacteria, and Bacteroidetes phyla; five anaerobic bacterial genera were identified as being associated with PCa risk groups.
Tsai et al.	2022	16S rRNA sequencing technology	Urine samples from 62 PCa patients, 77 BPH patients, and 46 normal men	The microbial composition of urine samples from BPH and PCa patients showed significant differences compared to the control group; the genera Faecalibacterium, Staphylococcus, Neisseria, and Agathobacter were enriched in the PCa urine samples.
Gonçalves et al.	2023	16S rRNA sequencing technology	Urine and glans samples from 15 PCa patients and 15 normal men	The microbial community composition of urine and glans samples was similar; in PCa urine samples, the relative abundance of Firmicutes, Clostridia, Streptococcus, Prevotella, and Peptostreptococcus was elevated; in normal urine samples, the relative abundance of Proteobacteria, Betaproteobacteria, Methylobacterium, Faecalibacterium, and Blautia was higher.
Akinpelu et al.	2024	Bacterial culture and identification technology	Urine samples from 66 PCa patients and 40 BPH patients	The pathogen isolation rate was higher in PCa samples than in BPH samples, with Gram-negative bacteria predominating over Gram-positive bacteria. Escherichia coli was the most common, followed by Pseudomonas aeruginosa and others.

## 4 The role of *C. acnes* in PCa progression

Among the various pathogens investigated for their potential involvement in PCa, HPV remains the only microorganism with a well-established carcinogenic role. However, other bacteria—including *C. acnes*, *Escherichia coli*, and *Neisseria gonorrhoeae*—have been implicated in contributing to chronic inflammation associated with PCa pathogenesis (Lawson and Glenn, 2022). *C. acnes*, a common skin commensal, has recently garnered substantial attention for its role in promoting PCa progression through the induction of chronic inflammation. Multiple studies have reported that prostate host cells respond to *C. acnes* infection by secreting pro-inflammatory cytokines and chemokines, such as IL-6, IL-8, IL-10, and TNF-α (Davidsson et al., 2016; Kim et al., 2024). These inflammatory mediators are known to activate key oncogenic signaling pathways, including NF-κB, STAT3, and cGAS-STING axis (Fassi Fehri et al., 2011). Moreover, different subspecies of *C. acnes* possess distinct surface structures, which influence their tissue colonization capacity and pathogenic potential at both cutaneous and non-cutaneous sites (Mak et al., 2013a). Sahdo et al. demonstrated that *C. acnes* activates caspase-1 in human peripheral neutrophils, potentially initiating inflammation-driven prostate pathology (Sahdo et al., 2013). In support of this, Bae et al. performed immunohistochemical analyses and found *C. acnes* to be frequently present in non-cancerous glandular epithelial cells and stromal macrophages in PCa tissues. The infection was associated with nuclear NF-κB activation in prostate cells, suggesting a mechanistic link between *C. acnes*-induced inflammation and tumor progression (Bae et al., 2014). Animal model studies have further validated these findings. In rat models of prostate infection, *C. acnes* induced chronic histological inflammation and persistent infection, reinforcing its pathogenic potential in prostate tissue (Olsson et al., 2012; Shinohara et al., 2013). Recent investigations have also revealed that *C. acnes* may activate immune responses through non-classical pathways. For example, Fischer et al. demonstrated that *C. acnes* can stimulate the cGAS-STING pathway in human macrophages, leading to IFN-I signaling and innate immune activation (Fischer et al., 2020). Additionally, Davidsson et al. reported a strong positive correlation between *C. acnes* presence and Treg infiltration in PCa tissues, suggesting that *C. acnes* may contribute to an immunosuppressive tumor microenvironment that facilitates cancer progression (Davidsson et al., 2021). Furthermore, Ashida et al. found that *C. acnes* infection altered the expression of genes involved in homologous recombination and the Fanconi anemia pathway in prostate epithelial cells. These changes impaired DNA repair mechanisms and induced a “BRCAness” phenotype (Ashida et al., 2024). This research highlights the potential role of *C. acnes* in driving PCa via both classical inflammatory and non-classical immune and genetic mechanisms.

**Figure 3:**
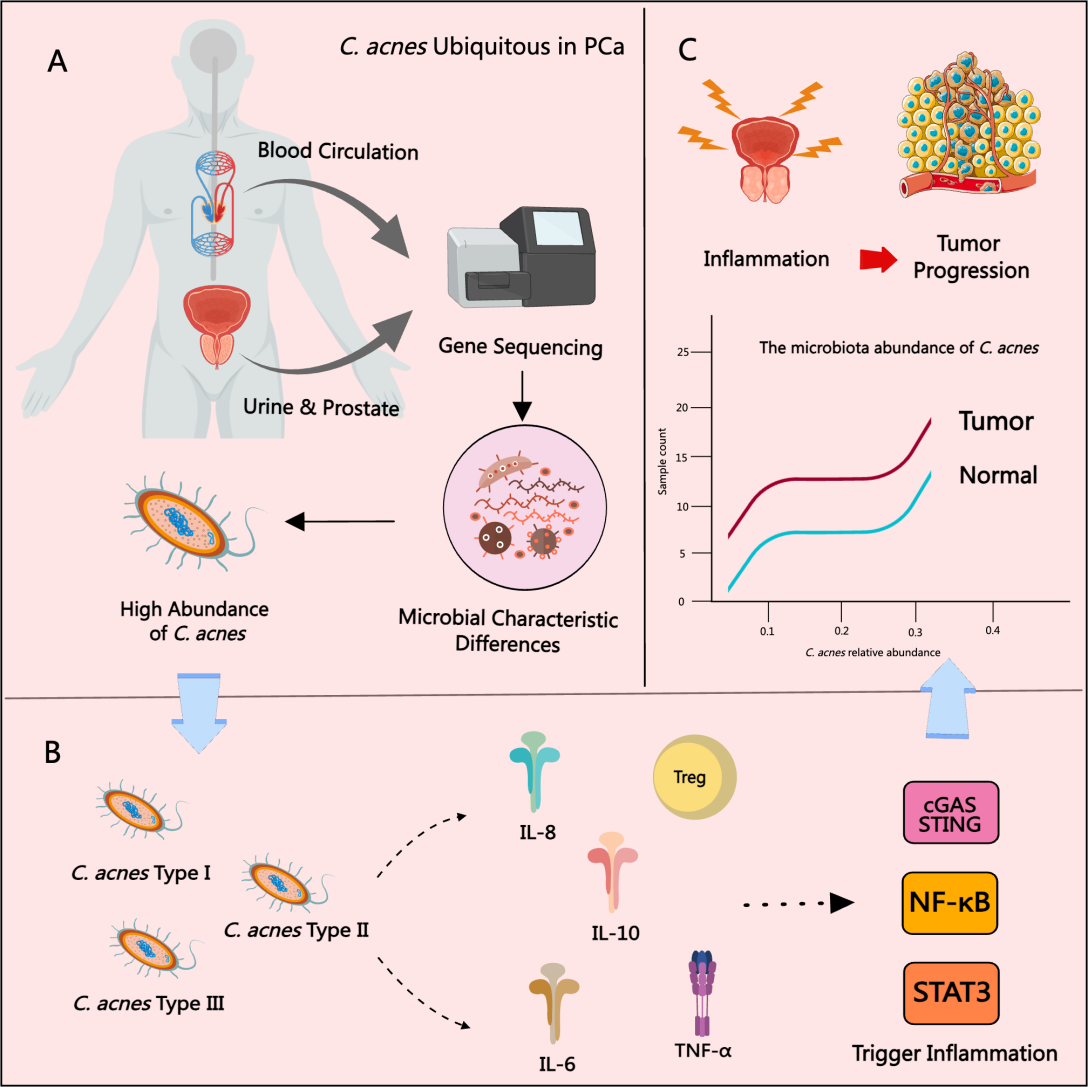
The role of *C*. *acnes* in PCa progression. **(A)** *C*. *acnes* is frequently detected in prostate tissue, urine, and blood from PCa patients. **(B)** The type II subtype of *C*. *acnes* is most strongly associated with PCa, promoting chronic inflammation by inducing inflammatory cytokines and chemokines and activating pathways such as NF-κB, STAT3, and cGAS–STING. **(C)** The relative abundance of *C*. *acnes* in PCa tissues is significantly correlated with increased tissue inflammation and higher tumor grade.

Extensive research has suggested that the C. acnes Type II subspecies—considered a more defensive phenotype—may be closely associated with the development and progression of PCa (Dekio et al., 2021). Notably, the prevalence of C. acnes Type II is higher in adult men than in adolescents, implying a potential age-related factor in its colonization. While Type I strains predominantly inhabit facial skin, Type II strains are more frequently detected in the urinary tract (Ba et al., 2006). In early studies, Cohen et al. cultured prostate tissue from PCa patients and identified *C. acnes* in 35% of the samples. The presence of *C. acnes* was significantly correlated with increased prostatic inflammation, and the strains isolated were genetically distinct from classical skin-associated strains (Cohen et al., 2005). Further evidence was provided by Mak et al., who used 16S rRNA gene sequencing and multi-locus sequence typing to confirm that prostate-isolated *C. acnes* strains were affiliated with sequence types typical of the urethral microbiota, differing from acne-associated skin strains (Mak et al., 2013b). Ba et al. detected *C. acnes* antibodies in patients undergoing prostate biopsy and found that elevated antibody titers were significantly associated with increased PSA levels. Antibody titers were also independently linked to patient age, prostate volume, and the degree of inflammation in BPH patients (Ba et al., 2008). Interestingly, Severi et al. reported an inverse relationship between circulating *C. acnes* antibody titers and PCa risk, particularly in advanced-stage cases (Severi et al., 2010). However, this study could not distinguish whether the elevated titers originated from acne-related colonization or later prostate infection, thus limiting the interpretation of causality. Chen et al. used RNA sequencing and detected *C. acnes* gene expression in both tumor and adjacent non-tumorous prostate tissue, but not in samples from healthy individuals (Chen and Wei, 2015), highlighting a potential role in early PCa pathogenesis. Davidsson et al., using bacterial culture and PCR, demonstrated that both Type IA and IB *C. acnes* were capable of colonizing the prostate, with significantly higher prevalence in PCa patients compared to controls (Davidsson et al., 2016). Subsequent studies have proposed that the presence of *C. acnes* in glandular tissue may serve as an independent early biomarker for PCa, potentially offering superior diagnostic value over PSA alone (Kakegawa et al., 2017). Additional evidence from Yow, Kim, Ahn, and Sarkar further supported the enrichment of *C. acnes* in PCa patients (Ahn et al., 2022; Kim et al., 2024; Sarkar et al., 2022; Yow et al., 2017). Notably, Kim et al. reported higher *C. acnes* abundance in high-grade PCa samples. LEfSe analysis identified *C. acnes* as a key microbial biomarker for distinguishing cancer, implicating it in PCa cell proliferation and inflammatory regulation (Kim et al., 2024). In addition to tissue-based studies, urinary microbiome analyses also demonstrated a significant increase in *C. acnes* among PCa patients (Ahn et al., 2022). Manente et al. detected *C. acnes* genomic DNA in urine and semen using real-time PCR, with no detection following antibiotic treatment (Manente et al., 2022). This suggests that *C. acnes* may promote the initiation and progression of PCa by triggering chronic inflammation and activating multiple signaling pathways, and can be detected through genomic analysis, as shown in **Figure 3**.

However, not all studies support the hypothesis that *C. acnes* promotes PCa progression through inflammatory responses. Alexeyev et al. conducted two independent studies using 16S rRNA sequencing and fluorescence *in situ* hybridization, identifying *C. acnes* as the most frequently detected bacterium in both BPH and PCa tissues. Their findings demonstrated that *C. acnes* is capable of establishing persistent infections within the prostate (Alexeyev et al., 2006, 2007). Similarly, Sfanos et al. screened a large number of prostate tissue samples for the presence of *C. acnes*, *Trichomonas vaginalis*, and other microbial DNA. While microbial DNA was detected in 87% of samples, culture-based methods isolated far fewer viable organisms, and no significant association was found between *C. acnes* presence and histological signs of inflammation (Sfanos et al., 2008). Additionally, a population-based survey study found no significant correlation between self-reported acne and PCa risk, suggesting that acne history cannot serve as a reliable biomarker for PCa susceptibility (Cremers et al., 2014). In a clinical cohort of 99 PCa patients, *C. acnes* was identified in 60 individuals; however, there were no significant differences in serum levels of inflammatory mediators between infected and non-infected patients. These findings imply that *C. acnes* may induce only localized, low-grade inflammation that does not manifest systemically, though it may still influence PCa onset and progression via microenvironmental modulation (Ugge et al., 2018a). Supporting this perspective, Radej et al. observed the presence of *C. acnes* in both PCa and BPH patients and proposed that the bacterium may modulate immune responses through Tregs (Radej et al., 2020). Cavarretta et al., using 16S rRNA sequencing, reported high *C. acnes* abundance not only in tumor tissues but also in peritumoral and non-tumorous prostate regions (Cavarretta et al., 2017), further suggesting that this bacterium may not be specific to malignant transformation. In line with this, Kim et al. found that *C. acnes* abundance was lower in high-grade tumors compared to intermediate-grade tumors, implying that the role of specific *C. acnes* subtypes in PCa may vary and remains to be clearly defined (Kim et al., 2024). Despite the lack of strong statistical associations, many researchers continue to explore the potential role of *C. acnes* in prostate inflammation and tumor development. For instance, a prospective cohort study by Ugge et al. revealed that severe acne during late adolescence may be linked to an elevated risk of PCa later in life (Ugge et al., 2018b). It is important to note that microbial contamination during sampling significantly affects detection reliability. As *C. acnes* is a common anaerobic skin commensal, strict adherence to sterile collection protocols is essential to avoid false-positive findings and ensure data integrity (Sfanos and Isaacs, 2008). Overall, although correlations between *C. acnes* and PCa have been observed, future investigations should utilize more precise and contamination-controlled methodologies to accurately determine the bacterium’s pathogenic role and clinical relevance.

## 5 Discussion and prospect

PCa is one of the most common and increasingly prevalent malignancies affecting men worldwide. In recent years, microbiome research has emerged as a promising frontier in oncology, offering novel insights and therapeutic possibilities for the diagnosis and treatment of PCa. However, compared to the relatively well-established research on the gut microbiome, investigations into the prostate tissue microbiome remain limited and underdeveloped. Existing studies have demonstrated that the microbiome within PCa tissues exhibits notable diversity and significant regional heterogeneity. These microbial communities may contribute to tumor initiation and progression by promoting chronic inflammation, particularly prostatitis. Furthermore, pronounced differences in microbial composition have been observed across populations of different ethnic and geographic backgrounds, suggesting that environmental exposures, dietary patterns, and host genetic factors may play critical roles in shaping the prostate microbiome. Importantly, the variability in current findings may also stem from methodological differences and issues related to sample contamination. Beyond bacterial taxa, the prostate-associated microbiome encompasses a broader spectrum of microorganisms, including viruses, fungi, and parasites. The interplay between these microbial constituents and host factors contributes to a complex and dynamic microecological system within the prostate tumor microenvironment. In parallel, urine and prostate fluid have been proposed as non-invasive alternatives for characterizing the prostate microbiome, particularly in PCa patients. Several studies suggest that the urinary microbiome may reflect disease-related microbial shifts and play a potential role in PCa pathogenesis. However, given anatomical and compositional differences—particularly in microbial abundance—between urine and prostate tissue, further validation is required to determine the reliability of urine as a surrogate marker for the prostate microbiome.

Among the various prostate-associated microorganisms, *C. acnes* has garnered considerable attention due to its stable enrichment within prostate tissues. Accumulating evidence indicates that *C. acnes* may contribute to the initiation and progression of PCa by inducing chronic inflammatory responses, suppressing local immune surveillance, and remodeling the tumor microenvironment. Although microbial population heterogeneity exists, multiple independent cohort studies have consistently observed the enrichment of *C. acnes* in PCa tissues, highlighting its potential as a broadly applicable biomarker. Moving forward, *C. acnes* holds promise as a microbial marker for early screening and monitoring of disease progression in PCa. Furthermore, precision antimicrobial strategies targeting *C. acnes* may offer novel avenues for adjunctive therapeutic interventions in PCa management. In conclusion, the relationship between PCa and the microbiome represents a complex and evolving field. It involves intricate interactions between diverse microbial populations and their regulation of immune, inflammatory, and oncogenic pathways within the tumor microenvironment. Future research should prioritize mechanistic investigations to elucidate how specific microbes influence PCa initiation and progression. Such efforts may lead to the identification of novel microbial biomarkers and therapeutic targets, paving the way for early diagnosis, personalized treatment, and microbiome-based interventions in PCa management.
